# Measuring the implementation of a group-based Lifestyle-integrated Functional Exercise (Mi-LiFE) intervention delivered in primary care for older adults aged 75 years or older: a pilot feasibility study protocol

**DOI:** 10.1186/s40814-015-0016-0

**Published:** 2015-05-31

**Authors:** Jenna C. Gibbs, Caitlin McArthur, James Milligan, Lindy Clemson, Linda Lee, Veronique M. Boscart, George Heckman, Carlos Rojas-Fernandez, Paul Stolee, Lora M. Giangregorio

**Affiliations:** 1 Department of Kinesiology, University of Waterloo, 200 University Avenue West, Waterloo, ON N2L 3G1 Canada; 2 Centre for Family Medicine-Family Health Team, Department of Family Medicine, McMaster University, 10B Victoria Street South, Kitchener, ON N2G 1C5 Canada; 3 Faculty of Health Sciences, University of Sydney, 75 East Street Lidcombe, Sydney, NSW 2006 Australia; 4 School of Health & Life Sciences and Community Services, Conestoga College, 299 Doon Valley Drive, Kitchener, ON N2G 4M4 Canada; 5 School of Public Health and Health Systems, University of Waterloo, 200 University Avenue West, Waterloo, ON N2L 3G1 Canada

**Keywords:** Chronic disease management, Exercise, Fall prevention, Older adults, Physical activity, Physical therapy, Primary care

## Abstract

**Background:**

Declines in function and quality of life, and an increased risk of cardiovascular events, falls, and fractures occur with aging and may be amenable to exercise intervention. Primary care is an ideal setting for identifying older adults in need of exercise intervention. However, a cost-effective, generalizable model of chronic disease management using exercise in a real-world setting remains elusive. Our objective is to measure the feasibility, potential effectiveness, and implementation of an evidence-based Lifestyle-integrated Functional strength and balance Exercise (LiFE) intervention adapted as a group-based format (Mi-LiFE) for primary care to promote increased physical activity levels in older adults aged 75 years or older. We hypothesize that the intervention will be feasible without modification if ≥30 individuals are recruited over 6 months, ≥75 % of our sample is retained, and ≥50 % of our sample complete exercises ≥3 days per week.

**Methods/design:**

A pre-post pilot study design will be used to evaluate feasibility, potential effectiveness, and implementation outcomes over a 6-month period in physically inactive older adults ≥75 years recruited from a local family health team practice. The reach, effectiveness, adoption, implementation, and maintenance (RE-AIM) framework will be applied to evaluate the public health effects of the intervention including outcomes both at the individual and organizational levels. A physical therapist will teach participants how to integrate strength and balance activities into their daily lives over one individual and four group-based sessions, and two phone calls. Assessments will be completed at baseline and 6 months. Feasibility outcomes include recruitment over 6 months, retention at follow-up, and adherence measured by activity diaries. Change in patient-centered and implementation outcomes that will be evaluated include physical activity levels using accelerometers and International Physical Activity Questionnaire, physical performance using short physical performance battery, quality of life using EQ5D questionnaire, falls and harms using daily calendar diaries and self-report, fidelity using descriptive feedback, barriers and facilitators to implementation using thematic content analysis, and process outcomes.

**Discussion:**

The feasibility and implementation of the Mi-LiFE intervention in primary care for older adults will be evaluated, as well as the effects of the intervention on secondary outcomes. If the intervention appears feasible, we will use the resultant information to design a larger trial.

**Trial registration:**

ClinicalTrials.gov: NCTO2266225

**Electronic supplementary material:**

The online version of this article (doi:10.1186/s40814-015-0016-0) contains supplementary material, which is available to authorized users.

## Background

Declines in physical performance and quality of life, and an increased risk of cardiovascular and other chronic diseases, falls, and fractures occur with aging and may be amenable to exercise intervention [1–3]. Physical inactivity is a well-known modifiable risk factor for comorbid conditions, hospitalization, disability, and mortality [4–6]. However, engaging older adults in traditional exercise is challenging, and substantial evidence demonstrates physical activity (PA) levels decline with age [7–9]. Community-based exercise programs may not meet the needs of older adults with chronic and complex conditions or be manageable because of physical or cognitive limitations or travel required. Although structured, supervised exercise can be effective, a lifestyle-focused group-based intervention may be more realistic to implement on a population-wide basis [2, 10–14]. Thus, there is a need to define how evidence-based exercise should be implemented in practice to engage older adults in chronic disease management and falls prevention.

Primary care is an ideal setting for identifying older adults in need of exercise intervention [15–19]. However, the delivery of individualized exercise prescription for all older adults may not be feasible. A cost-effective, generalizable model of chronic disease management and health promotion for older adults using exercise in this setting remains elusive [20–22]. Physical therapist (PT) or health promotor-led group-based exercise intervention delivered in primary care is a possible approach that may address resource constraints and facilitate physician referral. Recent research has shown that exercise in primary care may be cost-effective, but there is little research in older adults [15, 17, 23]. In addition, many effective exercise programs are not implemented in practice because of the resources required or other gaps in the research-to-practice continuum.

Previous pragmatic trials evaluating the implementation of primary care-based PA interventions in older adults have included exercise referral schemes [24, 25] or PA counseling [15, 21]. Exercise referral schemes, where a physician refers patients to an external health-care provider or community program, may increase PA levels in inactive older adults [25]. However, it is unclear whether this strategy promotes long-term adherence to exercise participation or if exercise referral schemes are safe and effective for frail elderly patients [24]. PA intervention where a physician or a nurse provides intensive PA counseling in interdisciplinary family health teams [15] demonstrated an increase in self-reported PA in healthy adult men and women compared to brief advice from a physician. Implementations of home- or facility-based exercise or falls prevention programs in primary care-based settings are also possible strategies to result in higher uptake and more sustainable PA [23, 26–29].

Randomized controlled trial (RCT) research demonstrates that exercise programs that include challenging balance and muscle strengthening exercises are effective for improving health outcomes and reducing falls [30, 1, 31, 32]. Recent work by Clemson et al. [1] demonstrated that teaching older adults how to integrate exercise into daily life activities over five home-based individual visits (the Lifestyle-integrated Functional Exercise (LiFE) program) was associated with an increase in self-reported PA, a 31 % reduction in the rate of falls, and improvements in balance and ankle strength compared to controls. The LiFE program is unique such that participants learn activities and then plan ways to integrate them into their day rather than perform them as part of a structured exercise program. An example of integrating the LiFE activities into a daily routine consists of doing a tandem walk on the way to the kitchen as a balance challenge [1]. The LiFE program is an alternative to traditional, structured exercise for increased PA and falls prevention with good retention and adherence rates in older adults aged 70 years or older [1, 30]. Therefore, implementing a group-based version of the LiFE program in a family health team practice (seen as a more efficient and realistic use of resources than individual delivery) is proposed as the next step in determining the feasibility and potential effectiveness of this strategy for chronic disease management and falls prevention. The findings from our pilot feasibility study will inform the feasibility of a future multi-center cluster RCT to evaluate the cost-effectiveness, safety, and effectiveness of a group-based version of the LiFE intervention and similar implementation studies of exercise intervention in primary care.

The rationale for the current study was to evaluate whether the LiFE intervention, which has been shown to be effective in reducing falls and increasing self-reported PA in a research setting, could be implemented in a real-world primary care practice. Since the original study [1] did not objectively measure whether the LiFE intervention increased PA levels in older adults, a secondary aim of this study was to evaluate whether the LiFE intervention increases PA levels measured by an accelerometer. Reach, effectiveness, adoption, implementation, and maintenance (RE-AIM) is an example of a multi-level evaluation framework that may facilitate the measurement of public health effects and barriers and facilitators to implementation [33]. Thus, we have designed a pilot pre-post feasibility study using the RE-AIM framework to measure the pragmatic implementation of a group-based version of the LiFE intervention, referred to as the Mi-LiFE intervention, in primary care for older adults aged 75 years or older. The Mi-LiFE intervention is proposed as a method to engage older adults in sustainable exercise participation and chronic disease management, and to increase PA levels, physical performance, and quality of life.

### Objectives and hypotheses

The overall aim of our pilot study will be to evaluate the feasibility, potential effectiveness, and implementation of a group-based version of the LiFE intervention in primary care for older adults aged 75 years or older using the RE-AIM framework (Table 1). Our primary objectives and hypotheses will be related to feasibility outcomes (recruitment, retention, and adherence) and include:

**Table 1:** Measurement of the implementation of the group-based Mi-LiFE intervention in primary care for older adults aged 75 years or older using the RE-AIM framework and evaluation dimensions

Dimension	Description	Study outcome	Level
Reach	Proportion of the target population that participated in intervention	• Number of participants recruited over 6 months—including data on eligibility and interest in program	Individual
Potential effectiveness	Effect of the intervention on specific individual outcomes	Primary outcome:	Individual
		• Change in moderate-to-vigorous physical activity levels (min/day) measured from pre- to post-intervention	
		Secondary outcomes:	
		• Change in self-reported physical activity levels (min/week) measured from pre- to post-intervention	
		• Change in composite SPPB score measured from pre- to post-intervention	
		• Change in EQ5D-3L five dimensions and VAS QOL score measured from pre- to post-intervention	
Adoption	Proportion of settings, practices, and plans that will adopt this intervention	• Number of physician or nurse referrals from each of the five pods within the family health team	Organization
Implementation	Extent to which the intervention is implemented as intended in the real world	• Fidelity to original clinical trial by Clemson et al. [1]	Organization
		• Barriers and facilitators reported by research staff, clinical team or participants during implementation	
Maintenance	Extent to which a program is sustained over time	• Retention at 6-month follow-up	Individual^a^
		• Adherence to the exercises at 6-month follow-up	

Modified from Glasgow et al. [33]

*SPPB* short physical performance battery, *EQ5D-3L* EuroQOL health questionnaire, *VAS* visual analogue scale, *QOL* quality of life

^a^Unable to evaluate maintenance at organization level because pilot follow-up length <12 months

1. to evaluate the number of participants we can recruit to participate in the program from a family health team practice over 6 months; the intervention will be considered feasible without modification if we recruit 30 participants over 6 months based on data collected from the family health team’s geriatric screening program.
2. to determine intervention retention rates; the intervention will be considered feasible without modification if 75 % of the sample completes the 6-month follow-up assessments. This criterion is based on the original LiFE study [1] wherein 78 % of participants completed the 6-month follow-up assessments.
3. to determine adherence to the intervention; the intervention will be considered feasible without modification if 50 % of the participants completes balance and strength activities ≥3 days/week over the 6-month study period. Our criteria are based on RCT data in exercise and falls prevention research wherein completion of balance and strength exercise ≥3 days/week was positively associated with PA and falls outcomes [1, 32, 34–36, 28].

Secondary objectives will evaluate the potential effectiveness of the intervention by measuring PA levels, physical performance, and quality of life, and implementation outcomes to inform a larger trial. Secondary outcome measures, such as PA levels measured using accelerometers or challenges recorded in the implementation log, will inform the future evolution of the research or implementation. Description of outcomes, hypotheses, outcome measures, and statistical analyses in the study protocol are shown in Table 2.

**Table 2:** Summary of outcomes, hypotheses, outcome measures, and methods of analysis

Outcomes	Hypotheses	Outcome measures	Methods of analysis
Feasibility			
Recruitment	Recruit ≥30 individuals over 6 months	• No. of participants recruited over 6 months	Descriptive statistics and estimates based on 95 % CI
Retention	Retain ≥75 % of our sample	• No. of participants who complete study visit 2	Descriptive statistics and estimates based on 95 % CI
Adherence	≥50 % of participants will complete exercises ≥3 d/week at 6 months	• Proportion of participants that complete exercises ≥3 d/week at 6 months	Descriptive statistics and estimates based on 95 % CI
Change in patient-centered outcome measures			
PA levels	Improvement from baseline to 6-month follow-up	• Moderate-to-vigorous, light, and sedentary activity via accelerometer	Paired *t* test
		• IPAQ	
Physical performance	Improvement from baseline to 6-month follow-up	• Composite SPPB score	Paired *t* test
		• Gait speed over 4 m	
		• Standing balance tests from SPPB	
		• Five-Times-Sit-to-Stand	
QOL	Improvement from baseline to 6-month follow-up	• EuroQOL EQ5D-3L dimensions	Paired *t* test
		• Visual analogue scale QOL score	
Eating habits	Improvement from baseline to 6-month follow-up	• TFEQ-R21 Items- subscale scores	Paired *t* test
Falls		• No. of self-reported falls on daily diary	Descriptive statistics and estimates based on 95 % CI
		• No. of fallers	
Harms		• Self-report no. of harms	Descriptive statistics and estimates based on 95 % CI
		• No. of injuries and hospital visits	
Physician/nurse acceptance		• No. of physicians/nurses that refer potential participants	Descriptive statistics and estimates based on 95 % CI
Barriers and facilitators to implementation		• Log filled out by research staff, clinicians, and participants	Thematic content analysis
Participant satisfaction with intervention		• Semi-structured interview	Thematic content analysis
Fidelity		• Fidelity evaluation of video-taped exercise sessions	Rating of compliance and descriptive feedback

*PA* physical activity, *IPAQ* International Physical Activity Questionnaire, *SPPB* short physical performance battery, *QOL* quality of life

## Methods/design

### Study design

We propose a pilot pre-post feasibility study of the Mi-LiFE intervention delivered in a family health team practice for older adults ≥75 years. The RE-AIM framework [33] will be used to measure the feasibility, change in patient-centered outcome measures, and implementation of the Mi-LiFE program in a real-world setting [37–39].

### Study setting

The study setting is a primary care-based family health team practice with four sites in the Kitchener-Waterloo area in Ontario, Canada. The family health team is comprised of family physicians, interdisciplinary health-care providers, support staff, and learners. There are five pods or teams of family physicians (two to five physicians per pod) that will refer potential participants for the current study. All study visits and intervention sessions will take place at the same site affiliated with the family health team practice.

### Recruitment

We plan to recruit a minimum of 30 individuals. There will be two recruitment modes: (1) the family health team geriatric screening program and (2) in-clinic physician or nurse referral. All patients ≥75 years without an acute illness presenting at the family health team practice over the 6-month recruitment period will be initially screened for eligibility through the geriatric screening program or in-clinic by a physician or nurse. For the first recruitment mode, potential participants will be screened for PA by a nurse and will be asked to choose which statement best describes their current activity status:

1. not physically active beyond moving around or walking during activities of daily living;
2. physically active occasionally or during certain seasons more than others;
3. physically active and participates in ≥30 min of moderate-intensity physical activities on ≥5 days/week.

Patients that are physically inactive (option 1) or occasionally physically active (option 2) will be targeted for the current study and told by the nurse or physician that they would benefit from more PA. Potential participants will then be asked if they would consent to their contact information being recorded to receive information about future exercise programs offered at the family health team practice. The research assistant will call these potential participants to describe the Mi-LiFE program and invite them to enroll. For the second recruitment mode, potential participants will be approached in-clinic by their physician or nurse to ask them whether they are interested in receiving more information about the Mi-LiFE program from the research assistant. If interested, the research assistant will be provided with the potential participant’s contact information and conduct similar procedures as the first recruitment mode. Physicians of interested participants will be notified and asked to assess whether their participant meets study eligibility criteria and to rule out exercise contraindications. Physicians will be provided with the American College of Sports Medicine list of absolute and relative exercise contraindications [40].

### Participants

Potential participants will be eligible for the study if they are: (i) ≥75 years and (ii) able to communicate and understand instructions in English. Individuals will be excluded from study participation if they are/have: (i) currently participating in a lower extremity strengthening and balance training program ≥3 days/week for ≥30 min/day, (ii) a known diagnosis of dementia, (iii) any significant lung disease, moderate-to-severe chronic obstructive pulmonary disease, and (iv) exercise contraindication(s) as determined by a physician. We will use a two-stage screening process to determine final clearance for the program and capacity to consent and participate. First, the potential participant’s physician will provide clearance for participation by reviewing eligibility for the study and signing a referral form. If the participant has cognitive impairment, the physician will confirm whether the potential participant would be able to participate if a caregiver attends the sessions and assists with program participation. Second, the participant will be required to recall the purpose and general structure of the program and their responsibilities following review of the information letter and consent form with the research assistant during study visit 1.

### Strategies to enhance recruitment

The exercise sessions will be held at one of the sites affiliated with the primary care practice based on the familiarity of the location and staff to the participants. If the participant has cognitive impairment, they will be required to attend the intervention sessions with a caregiver, who will assist them with the program. All participants will be encouraged to attend the program with a spouse/partner, caregiver, family member, or friend. If the spouse/partner, caregiver, family member, or friend is ≥65 years, they will be provided the opportunity to complete the assessments. Non-English speaking individuals will be eligible to participate in the program if they have access to a translator who can review the LiFE Participant’s Manual, attend the sessions, and participate in all aspects of the intervention. However, we are limited in our capacity to ensure that the translation is consistent with the intervention protocol.

### Study protocol

#### Study overview

Participants will complete baseline assessments (study visit 1) and then begin the intervention. The intervention includes attending one individual session (session 1) and four group-based sessions (sessions 2–5) with a PT in groups of five or fewer, to occur every 1–2 weeks, with follow-up phone calls at weeks 6 and 10. Participants will repeat the assessments at a 6-month follow-up visit (study visit 2). Refer to Table 3 for the schedule of enrollment, assessments, and intervention visits.

**Table 3:** Schedule of enrollment, assessments, and intervention

Activity	Staff member	Screening/consent	Study visit 1	Intervention	Study visit 2
Time-point			Baseline	10 weeks	6 months
Recruitment/screening					
Geriatric screening program/in-clinic referral	Physician or delegate	X			
Eligibility screening	Physician	X			
Information letter and informed consent	RA	X			
Assessments					
Medical health questionnaire	RA		X		X
Accelerometer	RA		X		X
IPAQ	RA		X		X
SPPB	RA		X		X
EuroQOL EQ5D-3L	RA		X		X
TFEQ-R21	RA		X	X^a^	X
Fidelity evaluation	Delegate			X	
Post-program/exit interview	RA			X^b^	X
Daily diary	RA			X	

*RA* research assistant, *IPAQ* International Physical Activity Questionnaire, *SPPB* short physical performance battery, *TFEQ-R21* Three Factor Eating Questionnaire-Revised 21 items

^a^Administer before exercise session 1

^b^Administer following exercise session 5

#### Study assessments

At study visit 1, the research assistant will provide an overview of the study and review the information letter and consent form with the potential participant. The research assistant will obtain written informed consent from all participants prior to study participation (Additional file 1- Participant information letter and consent form). Participants who do not consent to their data being collected for research purposes, but who have been referred to participate in the program, will be offered all aspects of the exercise program and materials, but will not have data collected.

The research assistant will conduct the baseline assessments including (1) demographic and medical history, (2) EuroQOL EQ5D-3L health questionnaire, (3) short physical performance battery (SPPB), (4) International Physical Activity Questionnaire (IPAQ), and (5) Three Factor Eating Questionnaire-Revised 21 (TFEQ-R21). Study visit 1 will last 75–90 min. Participants will be sent home with an accelerometer to wear for 7 days and will bring the monitor to session 1. Participants will receive the LiFE Participant’s Manual [41] as a resource for the intervention and a daily routine chart to complete and bring to session 1. Assessments will be repeated at 6-month follow-up (study visit 2). After the follow-up assessments are complete, a research staff member will obtain qualitative feedback on the intervention via a semi-structured interview in-person or by phone. Participants will be given the option to opt out of assessments or complete questionnaires by phone.

#### Intervention

The LiFE intervention involves teaching older adults how to integrate balance and strength activities into their daily lives at multiple times during the day [1]. Dr. Clemson, who designed the LiFE intervention in Australia and published on its efficacy [1], has agreed to collaborate on our study to test whether a group-based version of the intervention is pragmatic in practice. Dr. Clemson will provide access to the intervention materials. A PT will teach the LiFE balance and muscle strengthening principles and activities over five sessions and two follow-up phone calls (1 week and 1 month following completion of the final exercise session). For a more detailed description of the theoretical basis of the LiFE program and core underlying principles of balance and strength training, please refer to Clemson et al. [42]. Participants may discontinue the intervention at any time. Table 4 describes the schedule and outline of intervention visits and phone calls.

**Table 4:** Schedule and outline of the group-based Mi-LiFE intervention

Study session	Time-point	Delivery	Content
1	Week 1	Individual	• Physical therapist reviews daily routine chart with participant
			• Life assessment tool is completed to evaluate capacity to participate in intervention
			• Introduction to LiFE program; participants are provided with LiFE Participant’s Manual
			• Physical therapist teaches LiFE program—balance and strength training principles to participants
			• Physical therapist teaches one to two balance and one to two strength activities to be integrated into specific daily life activities
			• Participants learn to use activity planner
2	Week 2	Group	• Physical therapist teaches LiFE program and new balance and strength activities with an emphasis on progressing to more difficult variations of activities
			• Physical therapist teaches participants to integrate the activities into their daily tasks and encourage autonomy in selecting opportunities to embed activities
			• Participants complete activity planner and daily diary
3	Week 3	Group	See Session 2
4	Week 4	Group	See Session 2
5	Week 5	Group	See Session 2
Phone call 1	Week 6	Individual	• Physical therapist calls participants to provide support and encouragement
			• Physical therapist addresses challenges or barriers if present; reinforces successes
Phone call 2	Week 10	Individual	See Phone call 1

#### Setting and supervision

All intervention sessions will be held at the same location affiliated with the primary care practice. The PT will teach the activities to the participants in a setting similar to a home-based environment, which will be equipped with tables, chairs, couches, and a kitchen including countertop, cabinets/cupboards, and appliances. The PT that will deliver the intervention has been practicing for 3 years and has experience working with older adults in long-term care and community settings. The PT received training on the delivery of the LiFE intervention by reviewing the LiFE Trainer’s Manual [43]. The PT also implemented some of the LiFE activities into her daily routine, which provided personal experiences to share with the participants. Our approach to delivering the intervention is thought to be applicable to PTs, kinesiologists, and health promoters in family health teams or other primary care settings that may deliver the intervention per protocol using the LiFE Trainer’s Manual.

#### Session 1

The PT will meet with the participants individually during session 1. If a participant joins the program with a spouse/partner, family member, caregiver, or friend, both individuals may attend. The PT will review a 7-day daily routine chart with the participant to assess how, when, and where they can embed the LiFE activities into their daily routine [43]. The PT will administer the LiFE assessment tool, which evaluates musculoskeletal injury history and includes a 10-item balance and strength assessment. The PT will demonstrate each activity and record the level at which the participant can perform the activity (levels 0–4) [43]. The LiFE assessment tool will take 30 min to complete.

The PT will provide an overview of the LiFE program and describe the balance and strength training principles [41]. The PT will teach one to two balance and one to two strength activities, and instruct participants on how to document their plans and execution of the activities using an activity planner [43]. Participants will use the activity planner to identify the how, when, and where components for each activity, and establish a plan for embedding the LiFE activities in daily tasks. The PT will review the activity planner with the participants at subsequent sessions to identify successes and challenges with planning and progressing the activities.

The PT will teach participants how to self-monitor exercise participation using a daily calendar-style diary that asks:

1. Did you do any exercise today? If yes, respond to questions 2–4.
2. Did you do the LiFE strength or balance activities today? How many LiFE strength or balance activities did you do?
3. Did you do any other strength or balance exercises today (NOT taught in the LiFE program)?
4. How many minutes of aerobic PA did you do today? On a 0–10 scale (refer to the Borg Rating of Perceived Exertion Scale) [44, 45], how hard was it?

Adherence calendars will be reviewed at subsequent sessions and monthly thereafter. Session 1 will last 60–90 min.

#### Sessions 2–5

The PT will teach the program in groups of five or fewer. Participants will learn new balance and strength activities and will progress previously taught activities each session and plan how, when, and where to integrate the activities into the daily life activities [43]. By the end of session 5, the PT will have taught participants all of the balance and strength activities. Sessions 2–5 will last 60 min.

The PT will call the participants 1 week and 1 month following completion of session 5 and provide reinforcement and encouragement to perform the activities, discuss strategies to increase PA and progress the activities, and address any successes and challenges related to the intervention. The phone calls will last 15–30 min.

#### Data collection and management

Standard operating procedures, information letter and consent form, scripts, data forms, and checklists for visits and follow-up calls can be found in the Mi-LiFE study guide, version date August 18, 2014. Detailed instructions on data collection, checklists for visits and follow-up calls, and materials for outcome measures are outlined in the Mi-LiFE study guide. All research staff will review the Mi-LiFE study guide prior to the study start date. The research assistant will manage the study and perform the outcome assessments. Research staff will participate in training on recruitment, outcome assessment, and data management. The research assistant and PT will meet weekly to discuss the intervention delivery, and with the principal investigator or other research staff members as needed. A research staff member will enter data into an excel spreadsheet or statistical software database (e.g. Statistical Package for Social Sciences, SPSS Statistics, IBM, New York, USA). A meeting with the study investigators will be held to discuss program implementation as needed. Outcomes will be assessed at baseline (study visit 1) and 6 months (study visit 2). Recruitment, data collection, and intervention sessions for the trial are ongoing, and data cleaning and analysis has not begun.

### Outcomes

#### Feasibility

##### Recruitment, retention, and adherence

The primary outcomes are related to feasibility, including the number of participants that are recruited (recruitment) and retained (retention), and the number of days/week that the activities are completed (adherence). Recruitment will be defined as the number of participants recruited over 6 months, including participants who do not consent to their data being collected for research purposes. Retention will be defined as the number of participants who complete study visit 2 at 6-month follow-up. Adherence will be defined as the number of days/week that the participant integrates balance and strength activities into daily tasks and will be recorded daily on calendar-style diaries. Adherence will be 100 % if a participant completes balance and strength activities ≥3 days/week.

### Change in patient-centered outcome measures

#### PA levels

Participants will wear a commercially available accelerometer (ActiGraph GT3x, ActiGraph, Florida, USA) over the hip for 7 days, during waking hours and removing for bathing or swimming, following study visits 1 and 2. Participants will be asked to record the dates and times they wore the accelerometer on log diaries. The number of minutes spent in four intensity levels of activity (sedentary, light, moderate, and vigorous) will be determined using standard counts/minute-based intensity cut-points. The cut-points that will be applied are <100 for sedentary behaviors, 100–1041 for light activity, and >1042 for moderate-vigorous PA (a commonly used cut-point for older adults) [46–48]. Tri-axial data will be analyzed in 60-s epochs. Non-wear time will be identified and excluded if ≥60 min of continuous zeros [9]. Filters will be applied based on the dates and times the participant reports wearing the accelerometer. Only participants who wear the accelerometer for at least 4 days and at least 10 h/day will be included in analysis.

Participants will complete the IPAQ at study visits 1 and 2 to evaluate changes in self-reported time (e.g., number of sessions in the past 7 days, duration per session) spent in strength training, yoga/Tai Chi/other balance activity, vigorous PA, moderate PA, walking, and sitting/lying down awake [49]. Data from each activity will be summed to provide a total amount of time spent in PA over 7 days. Reliability and validity of the IPAQ has been reported [49]. Participants will also record daily exercise participation (aerobic PA duration and intensity, number of LiFE and non-LiFE strength and balance exercises completed) on diaries throughout the 6-month study.

#### Physical performance

Participants will complete physical performance tests at study visits 1 and 2. The SPPB [50] measures gait speed in a 4-m walk test, balance in side-by-side, semi-tandem and full-tandem positions [51, 52], and leg strength in the Five-Times-Sit-to-Stand test [53, 54]. Each performance test is assigned a categorical score ranging from 0 (inability to complete the test) to 4 (best performance). A summary score ranging from 0 (worst performance) to 12 (best performance) is calculated by summing the scores from the three performance tests.

#### Quality of life

Health-related quality of life will be assessed using the EuroQOL EQ5D-3L questionnaire at study visits 1 and 2. The EuroQOL EQ5D-3L questionnaire [55] asks the participant to indicate the statement that best describes their present health state with respect to mobility, self-care, usual activities, pain/discomfort, and anxiety/depression. A visual analogue scale will be administered separately, and the participant will be asked to indicate on a 0 to 100 scale (0 = worst imaginable health state to 100 = best imaginable health state) where they would rate their present health state in their own opinion. Evidence of construct validity [56] and good test-retest reliability [57] has been demonstrated.

#### Falls

Participants will be instructed to report whether or not a fall occurred by daily entry in calendar-style diaries throughout the entire 6-month study. A fall will be defined as “a slip or a trip where the person loses their balance and part or all of their body lands on the ground, floor, or lower level” [58]. A fall incidence form will be filled out by the research assistant or PT to document type of fall, location, date and time, injuries (if any), actions completed (e.g., medication, treatment, hospitalization, other), and health-care services use (e.g., physician visit, allied health care, home care, etc.).

#### Harms

Participants will be instructed by the research assistant to report adverse events (AE) or injuries (serious or otherwise) by phone or at intervention sessions. Participants will be asked about illnesses or injuries at follow-up phone calls and study visit 2. Intervention side effects (e.g., pain, falls, and injuries) and three types of AEs will represent secondary outcomes.

The three types of AEs include 1) serious adverse events (Health Canada definition—event that results in death, hospitalization, or disability); 2) events linked to intervention; and 3) events leading to study withdrawal or intervention cessation. If potential AEs are reported, an AE reporting form will be completed. If it is a fall, the fall incidence form will be completed first, and that information will be used to complete the AE reporting form. The principal investigator will review the forms. AEs must be reported to the research ethics boards. A two-member committee of researchers and clinicians not directly involved with data collection will review AEs after all participants complete the intervention and at study end. The committee will determine whether the events were due to the intervention and will have access to all data, and will inform on how we might move toward conduct of a larger-scale trial. There will be no stopping guidelines for the trial.

### Implementation

#### Barriers and facilitators to implementation

Barriers and facilitators to implementation reported by research staff, the PT, physicians/clinical staff, and participants will be recorded in an implementation log by a research assistant. The log entries, video-taped intervention sessions, intervention phone calls, and activity planners will be evaluated via thematic content analysis [59] to identify which aspects of the intervention were challenging and which outcome measures were difficult to complete.

#### Participant feedback on intervention

Qualitative feedback on the intervention will be obtained via semi-structured interviews with participants (in the presence of caregiver or translator as necessary) at completion of the intervention and at study visit 2. During the semi-structured interviews, we will cover a pre-set list of topics to assess reasons why individuals participated in the intervention, participant satisfaction with the intervention delivery and materials, benefits from participation, and adherence to and sustainability of the exercise program. If applicable, the effect of a caregiver or translator on feasibility (recruitment, retention, and adherence) and implementation outcomes (intervention satisfaction) will be evaluated.

#### Fidelity

Fidelity will be evaluated by auditing video-taped sessions (individual and group) for the first and last cohorts of participants (five or fewer individuals each cohort). Two independent reviewers not directly involved in data collection will review the video-taped sessions and provide feedback on the intervention fidelity using a checklist developed by our research team. The checklists will be compared for consistency, and a third reviewer will be used to resolve any discrepancies between reviewers. Written consent will be obtained from participants to video-tape the exercise sessions.

#### Descriptive and other data

Questionnaires will be used to collect demographic data, medical history (e.g., medical diagnoses, medication name, dose, and directions for use, history of falls in past 12 months, weight status history), and current health status at study visit 2. Height (cm) and weight (kg) will be used as anthropometric measures. Information on process outcomes will be collected to inform a future trial, including number of individuals eligible or ineligible for the study, number of individuals interested or not interested in participating, descriptive characteristics of those who do and do not agree to participate (e.g., age, sex), reasons why individuals declined participation, number of physician or nurse referrals from each of the five physician pods, cost to implement intervention, staff time and resources used during the intervention, and number of intervention visits attended. The TFEQ-R21 [60, 61] will be administered at study visit 1, prior to session 1, and study visit 2 to examine the change in three subscale dimensions related to eating habits: dietary cognitive restraint, uncontrolled eating, and emotional eating. A sub-study evaluating the reliability and relevance of this tool to measure eating habits in older adults will be conducted, and findings will be reported in a separate manuscript.

#### Strategies to enhance retention

Participants will primarily interact with the research assistant and PT during the intervention to build rapport. Participants will receive a phone call reminder from the research staff at least 1 day prior to each session, which will provide participants or caregivers with an opportunity to communicate continued interest in study participation or report any concerns (e.g., safety, difficulties with the intervention). Participants will receive feedback on the results of their outcome assessments after completion of study visit 2.

#### RE-AIM framework

Definitions of the RE-AIM framework and detailed information on how each component is applied in this study are outlined in Table 1. All participants will be screened for PA levels, and reach (individual level) will be the number of participants recruited relative to those eligible. Representativeness of the participants will be assessed with respect to self-reported and accelerometer-measured PA levels (minutes/week) and descriptive characteristics (e.g., age, sex, current and past health conditions, medication use, and history of falls). Potential effectiveness or change in patient-centered outcome measures (individual level) will be assessed at 6-month follow-up and include change in accelerometer-measured PA (primary outcome) and self-reported PA, physical performance, quality of life, and eating habits (secondary outcomes). For adoption (organizational level), the number of physician or nurse referrals from each of the five pods within the family health team will be measured. Barriers and facilitators to implementation (organizational level) will be described during the intervention, after the final session, and at 6-month follow-up. Maintenance (individual level) will be a adherence rates. We are unable to evaluate maintenance at the organization level because our follow-up length is <12 months [33]. For a more comprehensive description of the RE-AIM evaluation model, refer to Glasgow et al. [33] and King et al. [62].

#### Sample size calculations

Since this is a pilot study, the primary objective is to determine the feasibility of implementing the Mi-LiFE program in primary care and potential effectiveness at increasing PA levels. We will determine the number of participants that we can recruit over 6 months to inform the number of centers and time needed to achieve the required sample. Data collected from the participating family health team’s geriatric screening program over 6 months identified 198 individuals who were not regularly exercising and 59 of those individuals agreed to receive information about exercise programs. We anticipate 50 % of these individuals will agree to participate in our study based on data from Clemson et al. [1].

Accelerometer-measured PA is a proposed primary outcome for a larger-scale version of this pilot study. Thus, a sample size calculation was performed using G Power 3.1.2 (Universitat Kiel, Germany, 2009) to determine the number of participants needed to observe a statistically significant change from pre- to post-intervention in moderate-to-vigorous PA (minutes/day) (a secondary outcome in the current study). Based on evidence from primary care-based exercise interventions [63, 10, 15, 64, 65] and surveillance data in older adults [66, 67, 7], we need 26 participants (allowing for 20 % attrition) to detect a minimum difference (alpha = 0.05, 90 % power, effect size = 0.75) from pre- to post-intervention of a mean 15 min/day of accelerometer-measured moderate-to-vigorous PA with a standard deviation of 20 min/day.

#### Statistical analyses

The protocol was drafted in accordance with the SPIRIT 2013 Statement (www.spirit-statement.org) (Additional file 2 – SPIRIT 2013 Statement checklist). Reporting will be in accordance with CONSORT (www.consort-statement.org/). The RE-AIM framework will inform the analysis of study outcomes [33]. Participant characteristics and outcomes will be summarized using descriptive measures: mean (standard deviation) or median (min-max or interquartile range) for continuous variables; number (percent, %) for categorical variables; and absolute and percent change for longitudinal data. The primary and secondary statistical analyses that will be performed for the current study are reported in Table 2. Subgroup or sensitivity analyses will be used to compare feasibility outcomes in men vs. women and secondary outcomes in adherent vs. non-adherent participants. Multiple imputation will be used to impute missing data [68]. No interim analyses are planned. *P* values will be reported to three decimal places. Analyses will be performed with SPSS Statistics version 22 or more recent version (Armonk, New York, USA) or SAS version 9.2 or more recent version (Cary, North Carolina, USA).

#### Ethics and confidentiality

The research will be conducted according to the Tri-Council Policy Statement, second edition (http://www.ethics.gc.ca/eng/policy-politique/initiatives/tcps2-eptc2/Default/). The study has received clearance from the University of Waterloo and Hamilton Integrated Research Ethics Boards. We received approval from the University of Waterloo Research Ethics Board to conduct a pilot test of the intervention sessions (one individual and two group sessions) in four individuals in our research space. Protocol amendments were made after initial ethics approval and pilot testing and have been approved by both ethics boards. Future amendments will be submitted to the ethics boards by the research assistant or principal investigator and updated in the registered protocol. A protocol deviation log will monitor individual deviations from the study protocol. Participants will be assigned an ID to be used on all forms and in the data management spreadsheet. De-identified data will be stored in a secured area at the study site. Hard copies of records with personal identifiers will be kept separately from the data. A research staff member will enter data into the data management system. Only the research assistant, PT, principal investigator, and data custodian will be able to view both the participants’ data and identifiers spreadsheet. The principal investigator or delegate will perform audits of the trial dataset and protocol deviations. The complete dataset will reside at the University of Waterloo. Research ethics boards will receive reports on and review all serious AEs and AEs related or possibly related to the intervention. Study results will be presented at conferences and published in academic journals. Authorship guidelines are outlined in the Mi-LiFE study guide, version date August 18, 2014. Professional writers will not be used.

#### Pre-pilot test of the Mi-LiFE intervention

We conducted a pre-pilot test of the intervention sessions (one individual session and two group sessions) with four individuals (three women and one man aged 76–85 years) in our research space. All intervention sessions were video-taped to evaluate therapist fidelity and delivery of the intervention. A delegate of Dr. Clemson reviewed and provided qualitative fidelity feedback on the video-taped sessions. Qualitative feedback on the intervention and related materials was obtained from all participants via semi-structured interviews at completion of group session 2. The research assistant and PT recorded successes and challenges faced during the pre-pilot test.

Tables 5 and 6 present the fidelity data from the pre-pilot test of the Mi-LiFE intervention. The PT received positive feedback on her explanation of the purpose of the program, and how she taught the balance and strength principles, and introduced the manual. The PT became increasingly capable of determining situations in which to embed the activities. The PT adapted the activity planner to a group-based setting and practiced tailoring the activities to participants’ abilities and providing individualized activity progression. Participants were able to share feedback on the program experience, problem-solve barriers to performing the activities, and reinforce their successes using the group-based format.

**Table 5:** Fidelity feedback on pre-pilot test of Mi-LiFE intervention in four participants—individual exercise session

Intervention element	Fidelity feedback comments
Purpose and aims of the LiFE program explained	“Explained manual and program. Overall quite well done.”
Daily routine chart used to plan how, when, and where activity will be performed and embedded	“Using the daily routine chart is not just about finding a ‘place’ in the home to do the LiFE activity, but a daily task or routine in which to embed the activity.”
LiFE assessment tool used to assess ability for each activity	“LAT was completed.”
Balance and strength principles taught and related to improving function and/or preventing falls	“The PT taught the principles well.”
Teaching the activity	“When introducing the activity try to demonstrate—this done to varying levels.”
• PT teaches the LiFE strength and/or balance principles related to the activity	“It is challenging to teach participants something to embed in a daily task when you can’t demonstrate in the home. The PT got more imaginative as she went along.”
• PT demonstrates the activity and identifies situation(s) to embed activity	
• Participant performs activity and confirms/identifies additional situation(s) to embed activity	
• Participant technique corrected as needed	“Good technique correction for tandem stand.”
• Provide positive reinforcement and encouragement	
Appropriate number of activities and level of difficulty taught for participant’s ability	“Only 1 strength activity done—I think certain participants could have managed at least 2.”
Recording of plans for activity performance is done on activity planner with activities linked to daily task	“Planning and recording sheets—it is really important to reinforce why they need to do them. They assist in making the activities habitual.”
Familiarity with participant’s manual demonstrated by PT	“Introduction to manual. Try to link activities to where they do things (e.g., cooking, in the workshop). Most other principles and key ideas covered well.”
Key points of program explained and reinforced	“The PT could engage the participant in determining how, when and where the LiFE activity could be embedded.”
• Look for opportunities in daily tasks or routines	
• Embed activities	
• Change habits	
• Challenge yourself	
• Safety	
Other key points mentioned	“The one participant does a lot of sedentary activities. You can then try to build the LiFE activities into these.”
• Practice	
• Advance slowly	
• Modify environment to facilitate performance of activities	
• Build in prompts/situational or environment cues to remind to do activity	
Wrap-up: Participant and therapist decide on activities to perform independently and requirements until next session and how to record activities	“PT often said ‘I will give you …to do’. Perhaps, it would have been better to have the participants think about and verbalize what they might like to do/what they find challenging.”

*PT* physical therapist

**Table 6:** Fidelity feedback on pre-pilot test of Mi-LiFE intervention in four participants—group exercise sessions

Intervention element	Fidelity feedback comments
Review of activities since last visit (e.g., share successes/challenges with PT and other group members)	“Individual review of previous activities—done well. PT could have addressed the questions back to the group and made use of group problem-solving.”
	“Participants could share more. Each participant reported back, but group process could have been better utilized to have participants share and problem-solve with group rather than just with PT.”
Teaching the activity	“Try not to focus just on teaching the activities but get them to think about planning and embedding activities. Lots of the process is about having them think about how, when, and where they will embed.”
• PT teaches the LiFE strength and/or balance principles related to the activity	
• PT demonstrates the LiFE activity and identifies situation(s) to embed the activity	
• Participant performs activity and confirms/identifies situation(s) to embed the activity	“Remember to reinforce the principles while you are teaching an activity—bringing your feet closer together is decreasing your base of support. Do not just talk about bringing your feet closer—talk about principle and reinforce why it challenged balance.”
• Participant technique corrected as needed	
• Provide positive reinforcement and encouragement	
Appropriate number and progression of activities taught for participant’s ability	“It is important for them to understand how to do the activity properly so that when they upgrade they will more likely be safe.”
Key points of program reinforced	“The PT taught the key points well.”
• Look for opportunities in daily tasks or routines	“When the participant said ‘it just becomes habit’—it would have been a good opportunity to reinforce that this is a key concept of the program.”
• Embed activities	
• Change habits	“Do not tell them how to make it more challenging until they have the idea of how to do it and have started to embed it.”
• Challenge yourself	
• Safety	
Planning and recording of activities reinforced	“Good PT problem solving for activity planners… It is important to explain WHY they are using these tools.”
Wrap-up: PT explains/reinforces what activities to perform independently and requirements until next session	“The PT said ‘think of a place in the house where you can do it’. Get each of them to tell the group how, when, and where they will embed that activity.”
	“Although not compulsory, it would be good for participants to familiarize themselves with the manual between session 1 and 2. They are allowed to ‘read ahead’—they may find something that they particularly want to do—it is their program - not so therapist led.”

*PT* physical therapist

Participant feedback on the pre-pilot test of the Mi-LiFE intervention is presented in Table 7. Participants reported positive feedback on the group-based approach and responded that the manual was easy to understand and adequately supplemented the intervention. Participants were able to integrate the activities into their daily routine, and all four participants planned to continue to perform the activities taught in the program. Participants experienced challenges understanding the activity planner and recommended that the PT provide more demonstration and repetition of the activities.

**Table 7:** Feedback from the pre-pilot test of the Mi-LiFE intervention and related materials in four participants

Feedback questions	Comments
What did you like or dislike about the exercise program or the materials provided for you?	Liked:
	• “Preferred group program vs. individual program- because motivation is greater in a group”
	• “The manual with pictures and explanations”
	• “Exercises were interesting”
	• “Group format was fine”
	• “Exercises were simple and easy to fit into routine and complete as you are doing other activities”
	• “Can do the exercises at home”
	• “Seeing what other people are challenged with”
	• “Hearing others’ experiences”
	Disliked:
	• “No dislikes”
	• “Problems understanding the recording sheets”
	• “Would sometimes forget to do the exercises”
Related to your participation in the exercise program, what could we have done better?	• “Instruction and exercises integrated more into the sessions”
	• “More demonstration and repetition of the exercises”
	• “Exercise program was done quite well”
	• “The explanation of the activity counter could have been more detailed at first introduction”
	• “First time in an exercise program and did not have any past experience to compare it to”
	• “Check in from family doctor to stay accountable”
Will you continue to perform the activities taught in this study and integrate them into your activities of daily living? Why or why not?	• “At least some of them- there’s a lot to keep in mind”
	• “Hope so…time will tell…I will hopefully not forget”
	• “To improve balance”
Was the manual easy to understand? Why or why not?	• “Odd word I did not understand”
	• “Well done”
	• “Add sense of humor, cartoons—feeling or emotional component”
	• “It was easy to understand. Pictures were good”
	• “Yes, very easy”
Was the length of the manual appropriate? Why or why not?	• “A lot to read at one time, but could do with planning”
	• “Empty pages between chapters—didn’t know what they were for”
Was the wording of the manual appropriate? Why or why not?	• “Easy to understand”
	• “Might depend on the person”
	• “Think so. Made sense”
	• “Did not recall any specific words that were different”
	• “Some words were unusual”
What overall changes would you recommend to improve the manual?	• “Probably good right now”
	• “Place manual in a binder or duotang—able to turn pages more easily”
	• “Add page numbers to recording sheets [activity planner]”
Were the principles and key points of the LiFE program communicated clearly and effectively in the manual? Why or why not?	• “Well done—pictures, wording”
	• “Yes”
	• “Physical therapist gave good explanations and demonstrations. The one-on-one session was very helpful”
	• “Would not have understood exercises without the manual”
Were the instructions for the strength and balance exercises clear and easy to understand? Why or why not?	• “Found some exercises too difficult, add in progression”
	• “Yes, think so”
	• “Clear and straightforward”
Were the pictures helpful to provide demonstrations of the exercise? Why or why not?	• “Yes… And add cartoons, that’s funny”
	• “Very definitely”
	• “Demonstrate the exercises better”
	• “ Nice variety of models… made it more personal”
	• “Showed different levels of difficulty”

Several modifications were made to adapt the LiFE intervention for use in a group-based setting. The group-based version of the intervention was designed to encourage discussion among participants regarding any successes or challenges to performing the activities. In the Mi-LiFE intervention, the activities were planned as a group and ideas for how, when, and where to perform the activities were shared among participants and recorded using the activity planner. The PT facilitated peer-to-peer learning strategies and demonstrated the activities in the group setting to allow the participants to observe how others performed the activities. The PT provided individualized activity recommendations to the participants throughout the group sessions to accommodate variation among group members’ abilities. For the group-based version of the intervention, we reduced the number of forms participants were asked to complete to minimize the burden of diary completion and to optimize data collection about adherence, PA, and falls. The activity planner was revised to include page numbers in the manual related to each activity, a comments section to describe successes or challenges experienced performing the activities, and a section to record the initial plan and subsequent progressions for each activity.

## Discussion

The Mi-LiFE intervention was designed to address the increased risk of chronic diseases, falls, and fractures exacerbated by physical inactivity and aging, and the limited accessibility and resources for long-term maintenance of PA in older adults. Using a pragmatic evaluation framework, the current study will provide knowledge on the public health effects of implementing the Mi-LiFE intervention in a primary care practice. We are collecting data on challenges to implementation of the intervention and intended outcome measures to inform whether or not we move forward with a future larger-scale trial, and to identify possible major or minor modifications to our protocol. If the findings from our pilot study suggest that the trial may be feasible, a future definitive trial could adapt our protocol, and engage other family health teams provincially, or even nationally, to conduct a multi-center, cluster RCT with longer intensive intervention and follow-up. The objective of a larger-scale RCT would be to determine the cost-effectiveness, safety, and effectiveness of the Mi-LiFE intervention delivered in primary care for older adults, with accelerometer-measured PA as a primary endpoint.

The Mi-LiFE intervention addresses several limitations of previous primary care-based PA interventions for older adults. The Mi-LiFE intervention incorporates theory-driven behavioral strategies [69] and evidence-based exercise training elements [1] to promote higher uptake and adherence to exercise. The current protocol uses the RE-AIM framework to evaluate the reach, potential effectiveness, adoption, implementation, and maintenance [33] of the Mi-LiFE program in an interprofessional family health team practice. The eligibility criteria are consistent with a pragmatic trial [70, 71], such that we will include a wide range of participants to meaningfully assess the feasibility of and implementation for our trial. We will also evaluate the potential effectiveness of the Mi-LiFE program on patient-centered (PA, function) and health outcomes (falls).

A strength of our protocol is the measurement of PA levels using both accelerometers and self-report methods. Associations between self-reported PA and health outcomes are the basis of the PA recommendations for optimal health outcomes [72]. However, self-report measures often overestimate PA [73] and are a challenge for older adults to interpret [74, 49]. Accelerometer-derived PA levels are considered more accurate and may represent a favorable method of measuring PA in older adults [73]. Our study has the capacity to provide valuable pilot data on change in PA levels following the Mi-LiFE intervention and address research questions related to the agreement between accelerometer and self-report PA measures of screening and adherence in older adults.

Limitations of our pilot trial include the shorter lengths of recruitment and follow-up periods, which prevented us from examining the long-term maintenance of our program within the primary care context. Our findings related to adoption at the organization level are limited to our experience of implementing the intervention in one primary care practice with one expert deliverer. A multi-center cluster RCT is required to better evaluate acceptance and readiness for adopting the Mi-LiFE intervention in family health team practices, variation of implementation across deliverers and sites, and potential effectiveness of the intervention vs. a comparator.

In conclusion, findings from the Mi-LiFE study will inform a future definitive multi-center cluster RCT with a longer intensive intervention and follow-up. The current pilot study will evaluate feasibility and change in patient-centered outcome measures using the RE-AIM framework and offer insight on the implementation of an evidence-based exercise intervention in a real-world setting for physically inactive older adults.

## Trial status

ClinicalTrials.gov Identifier NCTO2266225 (Protocol: Mi-LiFE Study Guide, version date August 18, 2014; Trial Sponsor: Chronic Disease Prevention Initiative Seed Funding from the Propel Centre for Population Health Impact—University of Waterloo).

## Additional files

Additional file 1:
**Participant information letter and consent form.**

Additional file 2:
**SPIRIT 2013 Statement checklist: recommended items to address in a clinical trial protocol and related documents.**

## Abbreviations

AE: adverse events
TFEQ-R21: Three Factor Eating Questionnaire-Revised 21
LiFE: Lifestyle-integrated Functional Exercise program
Mi-LiFE: Measuring the implementation of the Lifestyle-integrated Functional Exercise program
PA: physical activity
PT: physical therapist
RCT: randomized controlled trial
RE-AIM: reach, effectiveness, adoption, implementation, and maintenance
SPPB: short physical performance battery

## References

[1] Clemson L, Fiatarone Singh MA, Bundy A, Cumming RG, Manollaras K, O’Loughlin P (2012). Integration of balance and strength training into daily life activity to reduce rate of falls in older people (the LiFE study): randomised parallel trial. BMJ..

[2] Pahor M, Guralnik JM, Ambrosius WT, Blair S, Bonds DE, Church TS (2014). Effect of structured physical activity on prevention of major mobility disability in older adults: the LIFE study randomized clinical trial. JAMA.

[3] Woolcott JC, Ashe MC, Miller WC, Shi P, Marra CA, Team PR (2010). Does physical activity reduce seniors’ need for healthcare?: a study of 24 281 Canadians. Br J Sports Med.

[4] World Health Report (2002). Reducing risks, promoting healthy life.

[5] Warburton DE, Nicol CW, Bredin SS (2006). Health benefits of physical activity: the evidence. CMAJ.

[6] Lee IM, Shiroma EJ, Lobelo F, Puska P, Blair SN, Katzmarzyk PT (2012). Effect of physical inactivity on major non-communicable diseases worldwide: an analysis of burden of disease and life expectancy. Lancet.

[7] Colley RC, Garriguet D, Janssen I, Craig CL, Clarke J, Tremblay MS (2011). Physical activity of Canadian adults: accelerometer results from the 2007 to 2009 Canadian Health Measures Survey. Health Rep.

[8] Ashe MC, Miller WC, Eng JJ, Noreau L (2009). Physical Activity Chronic Conditions Research Team. Older adults, chronic disease and leisure-time physical activity. Gerontology.

[9] Troiano RP, Berrigan D, Dodd KW, Masse LC, Tilert T, McDowell M (2008). Physical activity in the United States measured by accelerometer. Med Sci Sports Exerc.

[10] Pahor M, Blair SN, Espeland M, Fielding R, Gill TM, Life Study Investigators (2006). Effects of a physical activity intervention on measures of physical performance: results of the lifestyle interventions and independence for elders pilot (LIFE-P) study. J Gerontol A Biol Sci Med Sci.

[11] Dunn AL, Marcus BH, Kampert JB, Garcia ME, Kohl HW, Blair SN (1999). Comparison of lifestyle and structured interventions to increase physical activity and cardiorespiratory fitness: a randomized trial. JAMA.

[12] Nelson ME, Layne JE, Bernstein MJ, Nuernberger A, Castaneda C, Kaliton D (2004). The effects of multidimensional home-based exercise on functional performance in elderly people. J Gerontol A Biol Sci Med Sci.

[13] Delecluse C, Colman V, Roelants M, Verschueren S, Derave W, Ceux T (2004). Exercise programs for older men: mode and intensity to induce the highest possible health-related benefits. Prev Med.

[14] Opdenacker J, Delecluse C, Boen F (2011). A 2-year follow-up of a lifestyle physical activity versus a structured exercise intervention in older adults. J Am Geriatr Soc.

[15] Fortier MS, Hogg W, O’Sullivan TL, Blanchard C, Sigal RJ, Reid RD (2011). Impact of integrating a physical activity counsellor into the primary health care team: physical activity and health outcomes of the physical activity counselling randomized controlled trial. Appl Physiol Nutr Metab.

[16] Elley CR, Kerse N, Arroll B, Robinson E (2003). Effectiveness of counselling patients on physical activity in general practice: cluster randomised controlled trial. BMJ.

[17] Pinto BM, Goldstein MG, Ashba J, Sciamanna CN, Jette A (2005). Randomized controlled trial of physical activity counseling for older primary care patients. Am J Prev Med.

[18] Little P, Dorward M, Gralton S, Hammerton L, Pillinger J, White P (2004). A randomised controlled trial of three pragmatic approaches to initiate increased physical activity in sedentary patients with risk factors for cardiovascular disease. Br J Gen Pract.

[19] Jimmy G, Martin BW (2005). Implementation and effectiveness of a primary care based physical activity counselling scheme. Patient Educ Couns.

[20] Smith BJ, Bauman AE, Bull FC, Booth ML, Harris MF (2000). Promoting physical activity in general practice: a controlled trial of written advice and information materials. Br J Sports Med.

[21] Petrella RJ, Lattanzio CN (2002). Does counseling help patients get active? Systematic review of the literature. Can Fam Physician.

[22] Preventive US (2002). Services Task Force. Behavioral counseling in primary care to promote physical activity: recommendation and rationale. Ann Intern Med.

[23] Iliffe S, Kendrick D, Morris R, Masud T, Gage H, Skelton D, et al. Multicentre cluster randomised trial comparing a community group exercise programme and home-based exercise with usual care for people aged 65 years and over in primary care. Health Technol Assess. 2014;18(49):vii–xxvii. doi:10.3310/hta18490. 1–105. 10.3310/hta18490 PMC4781144 25098959

[24] Pavey TG, Anokye N, Taylor AH, Trueman P, Moxham T, Fox KR, et al. The clinical effectiveness and cost-effectiveness of exercise referral schemes: a systematic review and economic evaluation. Health Technol Assess. 2011;15(44):i–xii. doi:10.3310/hta15440. 1–254. 10.3310/hta15440 PMC4781450 22182828

[25] Pavey TG, Taylor AH, Fox KR, Hillsdon M, Anokye N, Campbell JL (2011). Effect of exercise referral schemes in primary care on physical activity and improving health outcomes: systematic review and meta-analysis. BMJ.

[26] Gardner MM, Buchner DM, Robertson MC, Campbell AJ (2001). Practical implementation of an exercise-based falls prevention programme. Age Ageing.

[27] Gardner MM, Phty M, Robertson MC, McGee R, Campbell AJ (2002). Application of a falls prevention program for older people to primary health care practice. Prev Med.

[28] Robertson MC, Devlin N, Gardner MM, Campbell AJ (2001). Effectiveness and economic evaluation of a nurse delivered home exercise programme to prevent falls. 1: Randomised controlled trial. BMJ.

[29] Ashworth NL, Chad KE, Harrison EL, Reeder BA, Marshall SC (2005). Home versus center based physical activity programs in older adults. Cochrane Database Syst Rev.

[30] Campbell AJ, Robertson MC, Gardner MM, Norton RN, Tilyard MW, Buchner DM (1997). Randomised controlled trial of a general practice programme of home based exercise to prevent falls in elderly women. BMJ.

[31] Sherrington C, Whitney JC, Lord SR, Herbert RD, Cumming RG, Close JC (2008). Effective exercise for the prevention of falls: a systematic review and meta-analysis. J Am Geriatr Soc.

[32] Campbell AJ, Robertson MC, Gardner MM, Norton RN, Buchner DM (1999). Falls prevention over 2 years: a randomized controlled trial in women 80 years and older. Age Ageing.

[33] Glasgow RE, Vogt TM, Boles SM (1999). Evaluating the public health impact of health promotion interventions: the RE-AIM framework. Am J Public Health.

[34] Campbell AJ, Robertson MC, La Grow SJ, Kerse NM, Sanderson GF, Jacobs RJ (2005). Randomised controlled trial of prevention of falls in people aged > or =75 with severe visual impairment: the VIP trial. BMJ.

[35] Hauer K, Rost B, Rutschle K, Opitz H, Specht N, Bartsch P (2001). Exercise training for rehabilitation and secondary prevention of falls in geriatric patients with a history of injurious falls. J Am Geriatr Soc.

[36] Suzuki T, Kim H, Yoshida H, Ishizaki T (2004). Randomized controlled trial of exercise intervention for the prevention of falls in community-dwelling elderly Japanese women. J Bone Miner Metab.

[37] Dzewaltowski DA, Estabrooks PA, Glasgow RE (2004). The future of physical activity behavior change research: what is needed to improve translation of research into health promotion practice?. Exerc Sport Sci Rev.

[38] Estabrooks PA, Bradshaw M, Dzewaltowski DA, Smith-Ray RL (2008). Determining the impact of Walk Kansas: applying a team-building approach to community physical activity promotion. Ann Behav Med.

[39] Gyurcsik NC, Brittain DR (2006). Partial examination of the public health impact of the People with Arthritis Can Exercise (PACE) program: reach, adoption, and maintenance. Public Health Nurs.

[40] American College of Sports Medicine (2013). ACSM’s guidelines for exercise testing and prescription.

[41] Clemson L, Munro J (2014). Lifestyle-integrated functional exercise program to prevent falls: participants manual.

[42] Clemson L, Singh MF, Bundy A, Cumming RG, Weissel E, Munro J (2010). LiFE pilot study: a randomised trial of balance and strength training embedded in daily life activity to reduce falls in older adults. Aust Occup Ther J.

[43] Clemson L, Munro J (2014). Lifestyle-integrated functional exercise program to prevent falls: trainers manual.

[44] Borg G (1962). Physical performance and perceived exertion.

[45] Borg G (1973). Perceived exertion, a note on “history” and methods. Med Sci Sports.

[46] Freedson PS, Melanson E, Sirard J (1998). Calibration of the Computer Science and Applications, Inc. accelerometer. Med Sci Sports Exerc.

[47] Copeland JL, Esliger DW (2009). Accelerometer assessment of physical activity in active, healthy older adults. J Aging Phys Act.

[48] Harris TJ, Owen CG, Victor CR, Adams R, Cook DG (2009). What factors are associated with physical activity in older people, assessed objectively by accelerometry?. Br J Sports Med.

[49] Craig CL, Marshall AL, Sjostrom M, Bauman AE, Booth ML, Ainsworth BE (2003). International physical activity questionnaire: 12-country reliability and validity. Med Sci Sports Exerc.

[50] Guralnik JM, Simonsick EM, Ferrucci L, Glynn RJ, Berkman LF, Blazer DG (1994). A short physical performance battery assessing lower extremity function: association with self-reported disability and prediction of mortality and nursing home admission. J Gerontol.

[51] Haines T, Kuys SS, Morrison G, Clarke J, Bew P, McPhail S (2007). Development and validation of the balance outcome measure for elder rehabilitation. Arch Phys Med Rehabil.

[52] Kuys SS, Morrison G, Bew PG, Clarke J, Haines TP (2011). Further validation of the balance outcome measure for elder rehabilitation. Arch Phys Med Rehabil.

[53] Meretta BM, Whitney SL, Marchetti GF, Sparto PJ, Muirhead RJ (2006). The five times sit to stand test: responsiveness to change and concurrent validity in adults undergoing vestibular rehabilitation. J Vestib Res.

[54] Whitney SL, Wrisley DM, Marchetti GF, Gee MA, Redfern MS, Furman JM (2005). Clinical measurement of sit-to-stand performance in people with balance disorders: validity of data for the Five-Times-Sit-to-Stand test. Phys Ther.

[55] Group EQ (1990). EuroQol—a new facility for the measurement of health-related quality of life.

[56] Brazier J, Jones N, Kind P (1993). Testing the validity of the Euroqol and comparing it with the SF-36 health survey questionnaire. Qual Life Res.

[57] van Agt HM, Essink-Bot ML, Krabbe PF, Bonsel GJ (1994). Test-retest reliability of health state valuations collected with the EuroQol questionnaire. Soc Sci Med.

[58] Buchner DM, Cress ME, Wagner EH, de Lateur BJ, Price R, Abrass IB (1993). The Seattle FICSIT/MoveIt study: the effect of exercise on gait and balance in older adults. J Am Geriatr Soc.

[59] Graneheim UH, Lundman B (2004). Qualitative content analysis in nursing research: concepts, procedures and measures to achieve trustworthiness. Nurse Educ Today.

[60] Stunkard AJ, Messick S (1985). The three-factor eating questionnaire to measure dietary restraint, disinhibition and hunger. J Psychosom Res.

[61] Cappelleri JC, Bushmakin AG, Gerber RA, Leidy NK, Sexton CC, Lowe MR (2009). Psychometric analysis of the Three-Factor Eating Questionnaire-R21: results from a large diverse sample of obese and non-obese participants. Int J Obes (Lond).

[62] King DK, Glasgow RE, Leeman-Castillo B (2010). Reaiming RE-AIM: using the model to plan, implement, and evaluate the effects of environmental change approaches to enhancing population health. Am J Public Health.

[63] Morey MC, Peterson MJ, Pieper CF, Sloane R, Crowley GM, Cowper PA (2009). The veterans learning to improve fitness and function in elders study: a randomized trial of primary care-based physical activity counseling for older men. J Am Geriatr Soc.

[64] Dubbert PM, Morey MC, Kirchner KA, Meydrech EF, Grothe K (2008). Counseling for home-based walking and strength exercise in older primary care patients. Arch Intern Med.

[65] Harris T, Kerry SM, Victor CR, Ekelund U, Woodcock A, Iliffe S (2015). A primary care nurse-delivered walking intervention in older adults: PACE (pedometer accelerometer consultation evaluation)-Lift cluster randomised controlled trial. PLoS Med.

[66] Evenson KR, Buchner DM, Morland KB (2012). Objective measurement of physical activity and sedentary behavior among US adults aged 60 years or older. Prev Chronic Dis.

[67] Loprinzi PD (2014). Accelerometer-determined sedentary and physical activity estimates among older adults with diabetes: considerations by demographic and comorbidity characteristics. J Aging Phys Act.

[68] Allison PD (2011). Missing data. Sage University papers series on quantitative applications in social sciences.

[69] Bleicher K, Cumming RG, Naganathan V, Travison TG, Sambrook PN, Blyth FM (2011). The role of fat and lean mass in bone loss in older men: findings from the CHAMP study. Bone.

[70] Thorpe KE, Zwarenstein M, Oxman AD, Treweek S, Furberg CD, Altman DG (2009). A pragmatic-explanatory continuum indicator summary (PRECIS): a tool to help trial designers. J Clin Epidemiol.

[71] Thorpe KE, Zwarenstein M, Oxman AD, Treweek S, Furberg CD, Altman DG (2009). A pragmatic-explanatory continuum indicator summary (PRECIS): a tool to help trial designers. CMAJ.

[72] Celis-Morales CA, Perez-Bravo F, Ibanez L, Salas C, Bailey ME, Gill JM (2012). Objective vs. self-reported physical activity and sedentary time: effects of measurement method on relationships with risk biomarkers. PLoS One.

[73] Dyrstad SM, Tjelta LI (2013). Newspaper coverage effects on the promotion of a lifestyle intervention program. J Obes.

[74] Topolski TD, LoGerfo J, Patrick DL, Williams B, Walwick J, Patrick MB (2006). The rapid assessment of physical activity (RAPA) among older adults. Prev Chronic Dis.

