# Supplementary material for: Measuring the implementation of a group-based Lifestyle-integrated Functional Exercise (Mi-LiFE) intervention delivered in primary care for older adults aged 75 years or older: a pilot feasibility study protocol
Source: Pilot Feasibility Stud. 2015 May 31;1:20. doi: 10.1186/s40814-015-0016-0 (PMC5154042; doi:10.1186/s40814-015-0016-0)
Supplement: Additional file 1: — Participant information letter and consent form. [file 40814_2015_16_MOESM1_ESM.pdf]

UNIVERSITY OF WATERLOO

McMaster University

Participant Information Sheet and Consent Form

Title of project: Feasibility of implementing a group-based lifestyle-integrated functional exercise (LiFE) intervention in primary care for older adults aged 75 years or older

Primary Investigator: Lora Giangregorio
Associate Professor
University of Waterloo, Department of Kinesiology
Tel: (519) 888-4567 Ext. 36357
Email: lmgiangr@uwaterloo.ca

Co-Investigators: Dr. Lindy Clemson, Dr. Jenna Gibbs, Dr. Linda Lee, Dr. James Milligan, Dr. Paul Stolee, Dr. Carlos Rojas-Fernandez, Dr. Veronique Boscart, and Dr. George Heckman

Student Investigator: Caitlin McArthur

Funding: University of Waterloo Propel Centre – Population Intervention for Chronic Disease Prevention Initiative

Introduction

You are being invited to participate in a research study. We have outlined detailed information about the study in this information letter and consent form, and will discuss it with you. Please read this information carefully and ask questions about anything you want to know more about.

Why is this research being done?

Physical activity has numerous benefits for older adults, including living longer, performing daily activities better, and improving quality of life. Integrating exercise into daily life activities may help to reduce falls and improve function in older adults. However, it is unclear how easy it would be to have a group-based program teaching older adults how to exercise linked to family doctor's offices in the real world.

What is the purpose of the study?

The purpose of this study is to evaluate how feasible it is to teach older adults aged 75 years or older how to integrate strength and balance exercises into their activities of daily living, in a group setting. Secondary purposes are to determine whether this

exercise program increases physical activity levels, and improves physical performance and quality of life in older adults. We aim to use the information obtained from this study to inform the development of a large community exercise program offered to older adults by their primary care provider.

Who qualifies for this study?

A screening process will take place over the phone to determine whether you qualify for the study (15 minutes). If you do not meet all of the criteria, you will not qualify for the study. To qualify for the study, you have to be: (i) over the age of 75 years, (ii) able to communicate in English (if you do not speak English, you may qualify if a translator can attend the program sessions with you and assist with completion of program materials), and (iii) able to give informed consent. You would not qualify if you: (i) currently participate in lower body strengthening and balance exercise 3 or more times per week for 30 minutes or more, (ii) have a known diagnosis of dementia, (iii) have any significant lung disease, moderate to severe chronic obstructive pulmonary disease (COPD), and (iv) have a chronic disease or condition that prevents you from exercising.

What will your responsibilities be if you decide to take part in the study?

If you volunteer to participate in the study, you will be asked to participate in an exercise program over a 6 month period at the Centre for Family Medicine in Kitchener, Ontario. You will be asked to come to the Centre for Family Medicine to complete study assessments at the start of the study and 6 months later at a follow-up assessment. We may ask you to describe the study back to us in your own words so we can be sure you understand what we are asking you to do. You will then attend one session with a physiotherapist where they will assess your balance and function. This session will last approximately 75 minutes. You will then receive a few exercises to try at home. You will be asked to attend four group exercise sessions led by a physiotherapist, 1-2 weeks apart, with follow-up phone calls at weeks 6 and 10. Phone calls will be audio-recorded to help us learn about and identify what you liked and did not like about the program. The group exercise sessions will last approximately 60 minutes.

Study assessments during study visits at the start and 6 months later:

- We will measure your physical activity levels using a small physical activity monitor that you will wear for 7 days on your hip during waking hours at the beginning and at the end of the study.

- You will be asked you to complete a questionnaire that will ask you about your physical activity levels in the past 7 days.

- A short physical performance assessment will be completed to assess your balance, walking speed over 4 meters, and leg strength. Balance tests include 1) standing with both feet together; 2) asking you to stand with your side heel of one foot touching the big toe of the other; and 3) standing with the heel of one foot in front of the toes of the other foot. You will be asked to stand still for 10 seconds for each condition. Leg strength will be assessed by how well you can get out of a chair 5 times. You can opt out of any of the tests if you are not comfortable doing them.

- You will be asked to complete 5 questions about pain, depression, mobility, self-care, and your usual activities. We will ask for permission to contact your physician if you indicate that you are depressed.

- We will ask a few questions about your medical history, such as health problems, medications, and history of falls or fractures. We will also ask if you consent to the researchers verifying your medical history with your physician.

- You will be provided with monthly diaries to keep track of your activity and whether you have had a fall.

- We may ask for your permission to videotape certain sessions of the exercise program.

- We will ask you for your feedback on the exercise program at the end of the program (around week 6) and at 6 month follow-up. We will audio-record these interviews to help us learn about and identify the successes and challenges of our exercise program.

What are the possible benefits of the study for me and/or society?

We cannot promise any personal benefits to you from your participation in the study. However, you will be provided with an exercise program that teaches you how to integrate strength and balance exercises into your activities of daily living. You will work with a physiotherapist to make sure the program meets your individual needs and hopefully increase your physical activity, reduce your risk of falling and improve your physical function. This study will provide valuable information that will be used to inform the development of an inexpensive and effective exercise program that can be delivered in the community or family doctor's offices.

What are the possible risks and discomforts?

All tests that are part of this study do not require you to perform any activity that you would not typically perform during your normal day-to-day activities. The exception is the balance tests where we ask you to stand with your feet beside and in front of each other. This may increase your risk of falling more than you would encounter in your daily life. However, there will be at least one health professional and a member of research staff present to assist if you lose your balance. It is also possible that you may experience mild muscle soreness and other exercise-related outcomes, such as minor changes in blood pressure and heart rate.

How many people will be in this study?

We aim to recruit at least 30 people from the Center for Family Medicine over six months.

What information will be kept private and confidential?

Your data will not be shared with anyone except with your consent or as required by law. All identifying information will be removed from the data and will be replaced with an ID code. A key file linking the ID code with your name will be separate from the data and will be stored on a password protected computer. A hard copy of the key file and all anonymized forms and study materials will be stored in Dr. Giangregorio's locked office and lab space in Burt Matthews Hall Rooms 1014 and 1109 at the University of Waterloo or in a password-protected computer database. Paper, audio recordings, and electronic records will be retained for 7 years after the study is complete, and study data will be retained for 25 years. All electronic data at rest and in transit will be password-protected. Only the research team will have access to the data. Some of the data will be used for student research projects, but your name or other identifying information will not appear with the data. Data will be secured in accordance with University of Waterloo policies available at http://ist.uwaterloo.ca/security/policy/.

Your health records and any information obtained during the research project are subject to inspection (for the purpose of verifying the procedures and the data) by the relevant authorities and authorized representatives of the University of Waterloo or as required by law. By signing the Consent Form, you authorize release of, or access to, this confidential information to the relevant study personnel and regulatory authorities as noted above.

It is anticipated that the results of this research project will be published or presented in a variety of forums. The results will be presented in such a way that you cannot be identified, except with your permission. You may be asked if you would consent to being video taped or having your photo taken during study activities for use in oral presentations, training information or publications. This is voluntary and not a requirement of the study. If you are to be video taped or photographed you will be asked to sign a separate consent form. Information about your participation in this research project may be recorded in your health records.

If I do not want to take part in the study, are there other choices?

It is important for you to know that you can choose not to participate in the study. Choosing not to participate will in no way affect the regular treatment or health care that you receive at the Center for Family Medicine. You are reminded that if you do not wish to sign this consent form to have your data collected for research but would still like to participate in the exercise program, you will receive all of the same services. However, you will not be asked to complete the study questionnaires, wear the activity monitor or record your daily activity or falls if they occur.

Can I end my participation early?

Participation in this research is voluntary. If you do not wish to take part, you do not have to. You will receive the best possible care whether or not you take part. If you choose to participate in this study, you may withdraw at any time. If you withdraw, you will be asked if there are some parts of the study you are still willing to complete (e.g., 6 month follow up study visit). We will not withdraw partially collected data unless you request that we do. The investigators may withdraw you from this research if circumstances arise which warrant doing so. If you decide to withdraw from the project, please notify a member of the research team.

Will I be paid to participate in this study?

You will not be paid to participate in the study. We will provide you a token for the parking lot at the Centre for Family Medicine on the days you come in for the study.

Are there any costs to me for my participation?
Your participation in this research project will not involve any additional costs to you or your health care insurer.

What happens if I have a research-related injury?

If you are harmed as a direct result of taking part in this study, all necessary medical treatment will be made available to you at no cost. By signing this form you do not give up any of your legal rights against the investigators, sponsor or involved institutions for compensation, nor does this form relieve the investigators, sponsor or involved institutions of their legal and professional responsibilities. If you have any urgent medical problem, injury or illness that is related to your participation in this study or have any questions, concerns or would like to speak to the study team for any reason please call:

Day Emergency Number: Dr. Jenna Gibbs at 519-888-4567 ext. 38779

PARTICIPANT COPY

Consent of Participant

I have read the information presented in the information letter about a study being conducted by Dr. Giangregorio and colleagues at the University of Waterloo. I have had the opportunity to ask any questions related to this study, to receive satisfactory answers to my questions, and any additional details I requested. I am aware that I may withdraw from the study without penalty at any time by advising the researchers of this decision. This project has been reviewed by, and received ethics clearance through, the Office of Research Ethics at the University of Waterloo.

I was informed that if I have any comments or concerns resulting from my participation in this study, I may contact Dr. Maureen Nummelin, Director, Office of Research Ethics at 519-888-4567 ext. 36005 or by email at maureen.nummelin@uwaterloo.ca.

With full knowledge of all foregoing, I agree, of my own free will to participate in this study. I have been advised that I will receive a signed copy of this form

Name of Participant

____

Signature of Participant   Date

____   _____________

Person obtaining consent: I have discussed this study in detail with the participant. I believe the participant understands what is involved in this study.

____

Name, Role in Study   Signature   Date

Name of Translator, if applicable   *Language translated into*

________________________________   ____________________________

Signature of Translator   *Date*

____________________________________   _____________

INVESTIGATOR COPY

Consent of Participant

I have read the information presented in the information letter about a study being conducted by Dr. Giangregorio and colleagues at the University of Waterloo. I have had the opportunity to ask any questions related to this study, to receive satisfactory answers to my questions, and any additional details I requested. I am aware that I may withdraw from the study without penalty at any time by advising the researchers of this decision. This project has been reviewed by, and received ethics clearance through, the Office of Research Ethics at the University of Waterloo.

I was informed that if I have any comments or concerns resulting from my participation in this study, I may contact Dr. Maureen Nummelin, Director, Office of Research Ethics at 519-888-4567 ext. 36005 or by email at maureen.nummelin@uwaterloo.ca.

With full knowledge of all foregoing, I agree, of my own free will to participate in this study. I have been advised that I will receive a signed copy of this form

Name of Participant

____

Signature of Participant Date

____ ___________

Person obtaining consent: I have discussed this study in detail with the participant. I believe the participant understands what is involved in this study.

____

Name, Role in Study Signature Date

Name of Translator, if applicable *Language translated into*

____________________________ ________________________

Signature of Translator *Date*

_______________________________ ___________
